# Abscess on the nasal tip of a young woman

**DOI:** 10.1016/j.jdcr.2025.01.042

**Published:** 2025-03-19

**Authors:** Ning C. McKenzie, Payton L. Smith, Mitchell Hanson, Jack P. Cossman

**Affiliations:** a Mayo Clinic Alix School of Medicine, Scottsdale, Arizona; b Department of Dermatology, University of California, San Francisco, California; c Medical College of Georgia, Augusta, Georgia; d Upper West Side Dermatology, New York, New York

**Keywords:** cutaneous abscess, LGBTQ+ health care, sexual orientation risk factors, sexually transmitted infections

## Patient presentation

**Fig 1:**
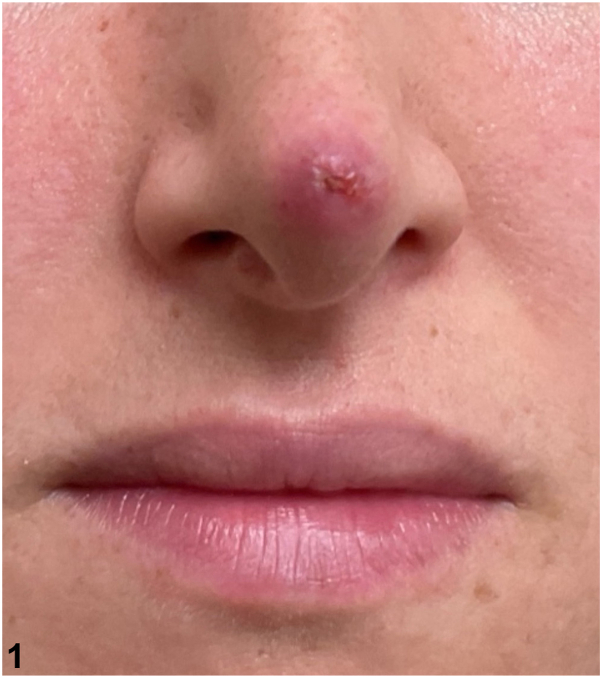

A 30-year-old female presented with a 6-month history of a persistent lesion on her nasal tip. The lesion failed to respond to a 4-week course of doxycycline 100 mg twice daily, a 2-week course of cephalexin 500 mg twice daily, and 2 injections of intralesional triamcinolone. The patient reported no significant past medical history and was sexually active with 1 female partner. On physical exam, the lesion appeared as a crusting, erythematous nodule with purulent discharge (Fig 1). Culture of the discharge grew 2+ gram-positive, catalase-negative, beta-hemolytic, Christie-Atkins-Munch-Petersen-positive, and facultatively anaerobic cocci arranged in chains.

**Question 1: What is the most likely mode of transmission of the causative organism?**

A. Direct skin-to-skin contact
B. Sexual transmission
C. Infected arthropod bite
D. Fomite transmission
E. Autoinoculation from asymptomatic colonization

**Answers:**

A. Direct skin-to-skin contact – Incorrect. While some *Streptococcus* species can be transmitted through skin contact, GBS is more often spread via mucosal or genital contact.^1^ Skin-to-skin transmission is less likely in this case, given the microbiologic findings and the patient's sexual history.
B. Sexual transmission – Correct. The patient has a localized abscess caused by Group B Streptococcus (GBS). GBS is commonly associated with genital and gastrointestinal tract colonization.^1^ Sexual transmission explains the chronicity of the nasal lesion, with a “ping-pong” effect due to reinfection between the patient and her partner. The partner's confirmed GBS colonization supports this mode of transmission.
C. Infected arthropod bite – Incorrect. Arthropod bites can become infected by pathogens such as *Staphylococcus aureus* or *Streptococcus pyogenes.* However, GBS is primarily associated with mucosal colonization and is not commonly linked to insect bites, making this explanation unlikely.
D. Fomite transmission – Incorrect. Although fomites can transmit certain infections, this is not a typical mode for GBS. The patient's lesion and sexual history strongly suggest direct, intimate contact, not indirect exposure through inanimate objects.
E. Autoinoculation from asymptomatic colonization – Incorrect. Airborne transmission is characteristic of respiratory pathogens like *Streptococcus pneumoniae*, not GBS. GBS is primarily spread through direct contact, particularly in the genital or mucosal regions, making this mode of transmission highly improbable.^2^ Oro-genital sexual transmission aligns with the patient's history, microbiologic findings, and the chronicity of the lesion.

**Question 2: During the sexual health history, the clinician asks, “Do you have any current partners? What genders are your partners?” The patient becomes hesitant and expresses concern about how the information will be used. What is the most appropriate response by the clinician to maintain an affirming approach?**

A. Reassure the patient that their information is needed solely to determine sexually transmitted infection (STI) risk
B. Apologize and move on, avoiding further discussion about their sexual history to preserve trust
C. Acknowledge the patient’s concern, explain the purpose of the questions in understanding health risks, and invite them to share as much as they feel comfortable
D. Ask if the patient has ever been tested for STIs and skip further details about their sexual practices
E. Validate the patient’s feelings, clarify that their responses will remain confidential, and recommend you discuss this at her next visit

**Answers:**

A. Reassure the patient that their information is needed solely to determine STI risk – Incorrect. Focusing solely on STI risk does not address the broader importance of sexual health and may make the patient feel their concerns about identity and confidentiality are not fully respected.
B. Apologize and move on, avoiding further discussion about their sexual history to preserve trust – Incorrect. Avoiding the discussion misses an opportunity to affirm the patient’s identity, address health concerns comprehensively, and build trust.^3^
C. Acknowledge the patient’s concern, explain the purpose of the questions in understanding health risks, and invite them to share as much as they feel comfortable – Correct. This response is culturally competent, acknowledges the patient’s hesitation, and transparently explains the medical relevance of the questions while empowering the patient to decide how much to share. It fosters trust and ensures the patient feels respected and in control.
D. Ask if the patient has ever been tested for STIs and skip further details about their sexual practices – Incorrect. Skipping details about sexual practices risks missing important health information and perpetuates assumptions, potentially compromising care.^3^
E. Validate the patient’s feelings, clarify that their responses will remain confidential, and recommend you discuss this at her next visit – Incorrect. Validating concerns and reassuring confidentiality are important, but this response does not fully explain the medical purpose of the questions nor allow her the chance to share today.

**Question 3: What is the most appropriate next step in management?**

A. Incision and drainage of the patient’s lesion and treating her partner with penicillin
B. Empiric treatment with oral terbinafine and obtaining a fungal culture
C. Treating the patient with topical mupirocin ointment and warm compresses
D. Performing a biopsy of the lesion and sending tissue for histopathologic examination
E. Treating the patient with oral clindamycin for gram-positive coverage

**Answers:**

A. Incision and drainage of the patient’s lesion and treating her partner with penicillin – Correct. Abscesses caused by GBS often require incision and drainage, as antibiotics alone may not suffice.^4^ Additionally, the patient’s partner is an asymptomatic carrier likely reinfecting the patient, so penicillin treatment is appropriate.^1^
B. Empiric treatment with oral terbinafine and obtaining a fungal culture – Incorrect. Although fungal infections can present as refractory nodules, the culture results showing beta-hemolytic, Christie-Atkins-Munch-Petersen-positive gram-positive cocci strongly indicate a bacterial, not fungal, etiology. Oral terbinafine is not warranted here.
C. Treating the patient with topical mupirocin ointment and warm compresses – Incorrect. While topical antibiotics can be effective for superficial bacterial infections, this patient’s chronic and refractory nodule suggests a deeper infection requiring more invasive management, such as incision and drainage and systemic antibiotic therapy.
D. Performing a biopsy of the lesion and sending tissue for histopathologic examination – Incorrect. A biopsy could be considered if there was suspicion of an atypical lesion (eg, malignancy or granulomatous infection). However, in this case, the diagnosis is clear based on the culture and clinical features, making biopsy unnecessary as the first-line step.
E. Treating the patient with oral clindamycin for gram-positive coverage – Incorrect. Although clindamycin provides effective gram-positive coverage, incision and drainage is critical in managing abscesses. Treating the partner is also essential to prevent reinfection, which clindamycin alone does not address.

## Conflicts of interest

None disclosed.
